# Comorbidity between Anorexia Nervosa and Autism Spectrum Disorder, a therapeutic challenge and worse prognosis? A case series

**DOI:** 10.1192/j.eurpsy.2025.1393

**Published:** 2025-08-26

**Authors:** P. Del Sol, A. Izquierdo, R. Fernández, M. García Moreno, J. Sanchez Cerezo

**Affiliations:** 1 Hospital Puerta de Hierro, Madrid; 2 Hospital Infanta Cristina, Parla, Spain

## Abstract

**Introduction:**

Anorexia nervosa (AN) and autism spectrum disorder (ASD) share symptoms that complicate diagnosis and treatment, including rigidity in thinking and behavior. This inflexibility often manifests as strict food routines in anorexia. Both conditions involve excessive preoccupation with control and perfection, leading to restrictive behaviors and heightened anxiety.

**Objectives:**

To show the clinical presentation of Anorexia Nervosa and austism spectrum disorder through the presentation of two cases.

**Methods:**

Cases presentation and literature review

**Results:**

Case 1

A 15-year-old girl is receiving psychiatric follow-up for restrictive eating habits. She developed normally and has used sophisticated language since childhood. However, during her infancy, she did not engage in symbolic play. Academically, she performs exceptionally well. She describes herself as having a very rigid personality and struggles with understanding irony and certain social behaviors. She has few friends, viewing friendships as a waste of time. Her interests lie deeply in literature and science, and she tends to wear childish clothing.

She expresses feelings of jealousy towards her younger sister and mentioned that she reduced her food intake to prevent growing taller and to achieve “thinner ankles.” As she begins psychotherapy, she shares that she finds it challenging to grasp what the psychologist means due to her tendency for literal thinking.

Case 2

The patient is a 14-year-old male from Peru, who arrived in Spain 8 months ago. Developmental milestones within the normal range. Little symbolic play and difficulty with non-verbal language. Highly ritualised behaviours and tendency to obsessions, requiring psychological intervention due to compulsion to clean in the COVID-19 pandemic. The patient was admitted to the inpatient unit for weight loss of 6 kilograms in one month, with food restriction and excessive increase in physical exercise. Selective mutism is associated with months of selective mutism, as ‘he does not speak to people who do not speak with a Peruvian accent’. Parents speak of a rigid and literal idea of ‘having to be thin in order to make friends and strong in order not to be weak’.

Both cases reflect how the rigidity and literal thinking of autistic disorder can lead to extreme behaviours such as ‘don’t eat so as not to grow up’ in the first case, or ‘don’t eat to make friends’ in the second case, lived with no flexibility

**Conclusions:**

There is a recognized connection between ASD and AN, with studies indicating a prevalence of 20-25%. Inflexible thinking associated with ASD can negatively impact the prognosis of AN, as ASD may contribute to the chronicity of the eating disorder. Additionally, psychotherapy can present challenges, and research suggests that behavioral techniques, particularly Applied Behavior Analysis (ABA), tend to yield better outcomes for individuals with AN.

**Disclosure of Interest:**

None Declared

